# Supplementary material for: Venomix: a simple bioinformatic pipeline for identifying and characterizing toxin gene candidates from transcriptomic data
Source: PeerJ. 2018 Jul 31;6:e5361. doi: 10.7717/peerj.5361 (PMC6074769; doi:10.7717/peerj.5361)
Supplement: Supplemental Information 3 [file peerj-06-5361-s003.gz › FinalOutput_E-6/L-amino-acid_oxidase_31/finaltree.pdf]

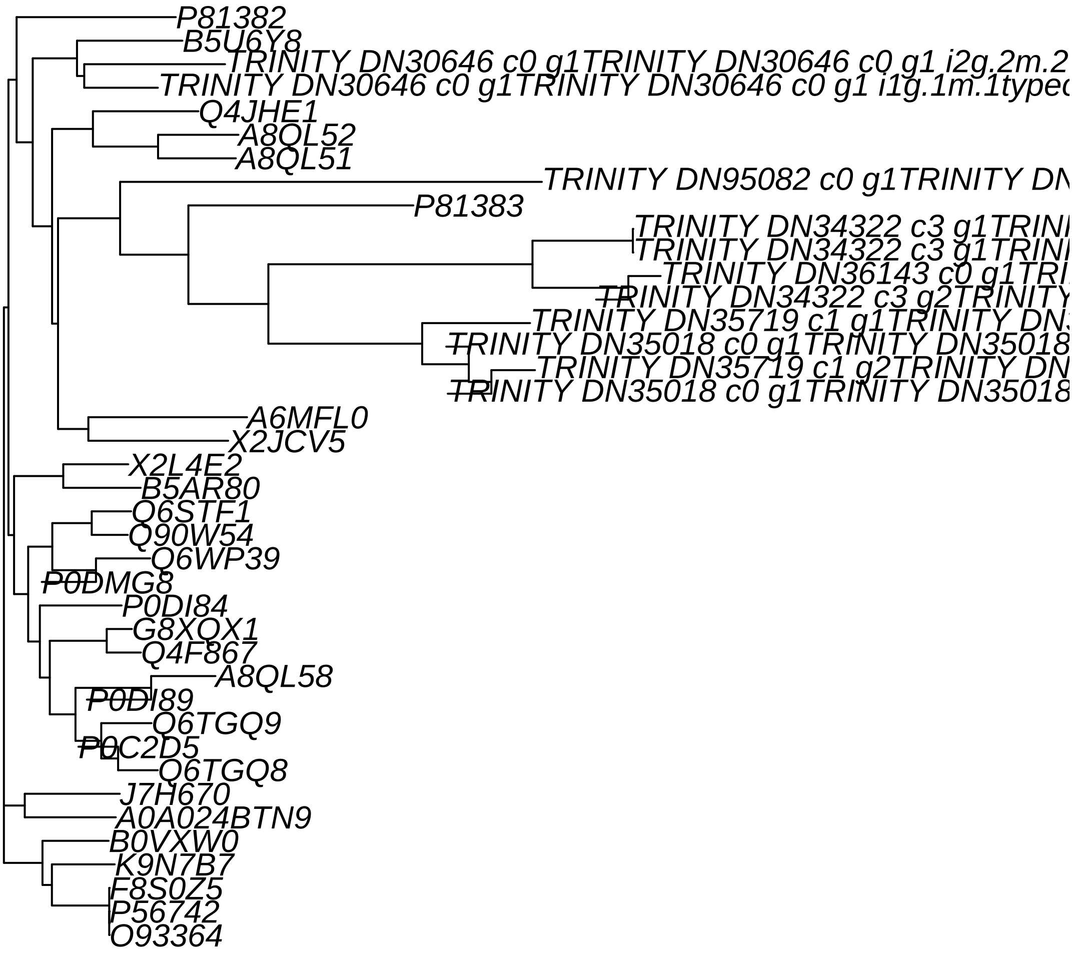
P81382
B5U6Y8
TRINITY DN30646 c0 g1TRINITY DN30646 c0 g1 i2g.2m.2
TRINITY DN30646 c0 g1TRINITY DN30646 c0 g1 i1g.1m.1typec
Q4JHE1
A8QL52
A8QL51
TRINITY DN95082 c0 g1TRINITY DN
P81383
TRINITY DN34322 c3 g1TRINI
TRINITY DN34322 c3 g1TRINI
TRINITY DN36143 c0 g1TRI
TRINITY DN34322 c3 g2TRINITY
TRINITY DN35719 c1 g1TRINITY DN3
TRINITY DN35018 c0 g1TRINITY DN35018
TRINITY DN35719 c1 g2TRINITY DN
TRINITY DN35018 c0 g1TRINITY DN35018
A6MFL0
X2JCV5
X2L4E2
B5AR80
Q6STF1
Q90W54
Q6WP39
P0DMG8
P0DI84
G8XQX1
Q4F867
A8QL58
P0DI89
Q6TGQ9
P0C2D5
Q6TGQ8
J7H670
A0A024BTN9
B0VXW0
K9N7B7
F8S0Z5
P56742
O93364
